# An Autopsy Case Report of an Infant Born to a Diabetic Mother, With Review of Literature—A Pandora's Box of Pathologies

**DOI:** 10.1155/crip/6662921

**Published:** 2025-10-26

**Authors:** Gurpreet Kaur, Raghav Sharma, Vikram Singh, Ankur Ahuja, Somasundaram Venkatesan

**Affiliations:** Department of Pathology, Armed Forces Medical college, Pune, India

**Keywords:** cardiac hypertrophy, case report, gestational diabetes, infant of diabetic mother (IDM), Pedersen hypothesis, pulmonary hypertension

## Abstract

Gestational diabetes mellitus (GDM) affects approximately 2%–17.8% of pregnancies, with preexisting diabetes also contributing to significant fetal risks. Despite advancements in obstetric and medical care, pregnancies complicated by maternal diabetes continue to carry a higher likelihood of fetal loss compared to nondiabetic pregnancies. Infants born to diabetic mothers (IDMs) are predisposed to complications such as birth trauma, respiratory difficulties, metabolic derangements including hypoglycemia and hypocalcemia, jaundice, increased blood viscosity, and various congenital anomalies—all potentially contributing to fetal mortality. We report the autopsy findings of a male IDM, born to a 25-year-old primigravida conceived via intrauterine insemination (IUI) with GDM, delivered at 38 weeks and 3 days of gestation, weighing 3.1 kg with reassuring Apgar scores at birth. Despite an apparently uncomplicated delivery and no immediate congenital anomalies detected, the infant developed sudden respiratory distress and unresponsiveness at 90 min of life, leading to unsuccessful resuscitation efforts. The autopsy revealed hallmark features of maternal–fetal glucose imbalance, including cardiopulmonary hypertrophy, hepatomegaly, immature lungs, pulmonary hypertension, and marked pancreatic islet cell hyperplasia. Inflammatory changes in the meninges and hypoxic neuronal injury were also observed. No structural malformations were identified. Although neonatal autopsies are inherently challenging, they provide critical insights that may influence neonatal care practices and guide future genetic counseling for affected families.

## 1. Introduction

Diabetes mellitus (DM) is a chronic metabolic disorder marked by impaired insulin production and/or action, affecting individuals across all age groups—from fetal life to adulthood. Among pregnant women, gestational diabetes mellitus (GDM) represents one of the most prevalent metabolic complications, impacting millions globally [1]. First described by [2], GDM is defined as hyperglycemia identified for the first time during pregnancy. With the global rise in obesity reaching epidemic proportions, the number of women diagnosed with GDM continues to increase, heightening the risk of various pregnancy-related complications [3].

The association between maternal diabetes and congenital malformations was recognized as early as 1885 when Lecorche reported hydrocephalus in infants born to diabetic mothers (IDMs) [4]. He identified hydrocephalus in two out of four infants exhibiting significant abnormalities, all born to diabetic mothers. GDM significantly elevates the likelihood of adverse outcomes such as fetal macrosomia, birth injuries, and increased rates of cesarean deliveries. However, these outcomes can vary based on factors including diagnostic criteria, screening protocols, the population studied, and socioeconomic conditions.

## 2. Case Report

A male neonate was delivered to a 25-year-old primigravida at 38 weeks and 3 days of gestation via spontaneous vaginal delivery in vertex presentation. The mother had a documented diagnosis of GDM, managed with metformin, and underwent regular antenatal glucose monitoring, which remained within acceptable limits. Conception was achieved through intrauterine insemination (IUI), confirmed by ultrasonography. Routine antenatal care and vaccinations were completed as per standard guidelines.

The newborn's birth weight was 3.1 kg, with Apgar scores of 7 at 1 min and 9 at 5 min. The infant cried immediately after birth, and a prominent caput succedaneum was noted over the right parietal region. Detailed physical examination revealed no dysmorphic features or evidence of birth trauma, and the perinatal transition was uneventful. Breastfeeding was successfully initiated.

Approximately 90 min postpartum, the infant became unresponsive. There were no signs of spontaneous breathing, cardiac activity, or palpable pulse, accompanied by multiple seizure episodes. Immediate cardiopulmonary resuscitation (CPR) was commenced, following which the neonate was transferred to the neonatal intensive care unit (NICU). Despite approximately 25–30 min of resuscitation, including mechanical ventilation and inotropic support, the infant could not be revived.

**Figure 1:**
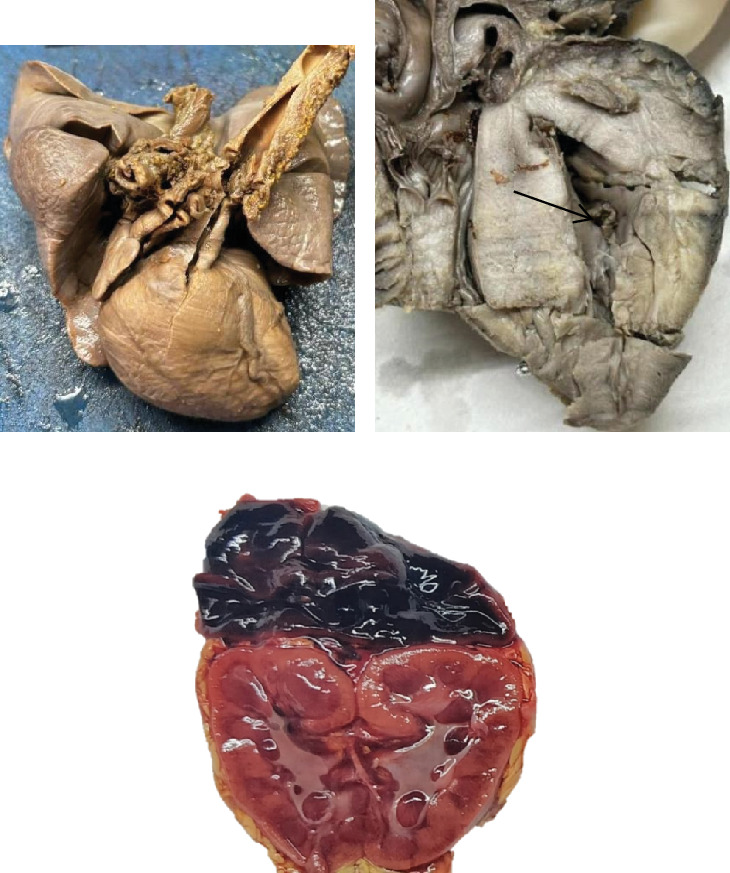
(a) Gross picture of the heart–lung complex showing straightening of the left heart border and rounding of the right heart border. (b) A four-chamber view shows thickening of the left ventricular wall, right ventricular wall, and interventricular septum, causing the left ventricular cavity to appear banana-like (black arrow). (c) Cut surface of kidneys and adrenals shows a grossly hemorrhagic adrenal.

**Figure 2:**
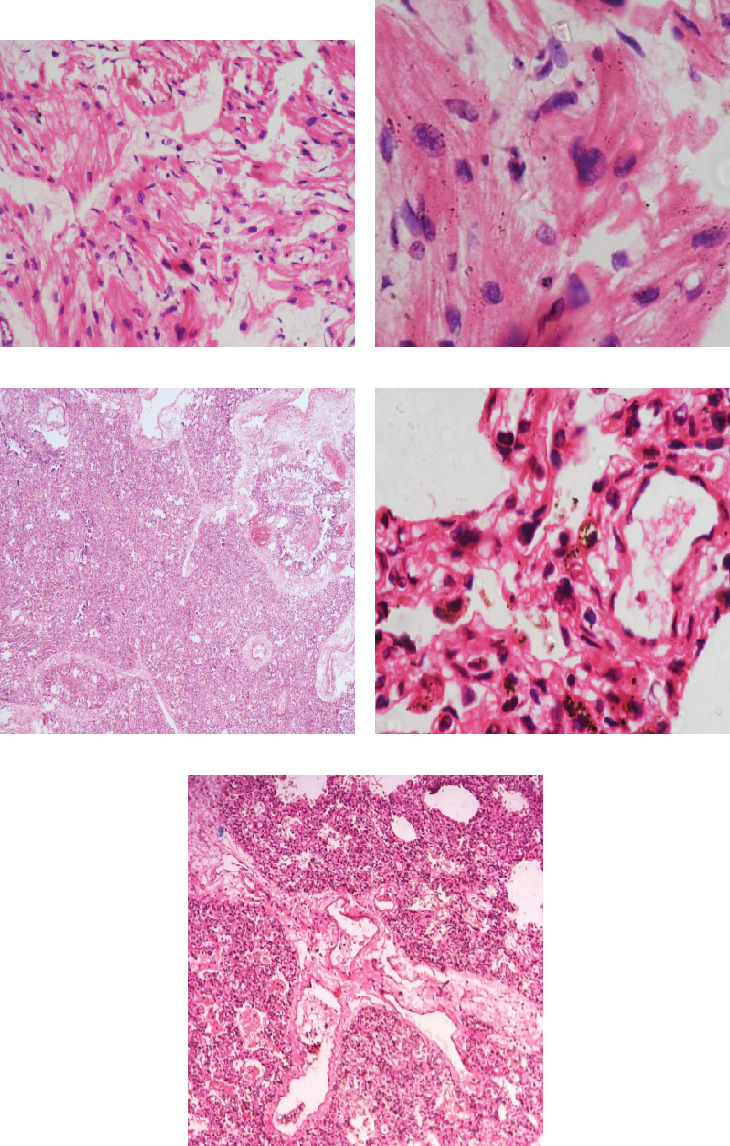
(a, b) Sections from cardiac tissue showing myofibrillar disarray in the heart, with fibers arranged perpendicularly and at oblique angles to each other. Nucleomegaly and a whirling pattern of the fibers may be appreciated at higher power. (c) High power view of the lung showed features of immaturity with thickened alveolar septae and angiomatoid vessels. Hemosiderin-laden macrophages were seen, indicating chronic venous congestion. Pulmonary vessels showed the presence of plexiform lesions. (d) Suggestive of pulmonary hypertension. (e) There was medial hypertrophy of the vessel walls.

**Figure 3:**
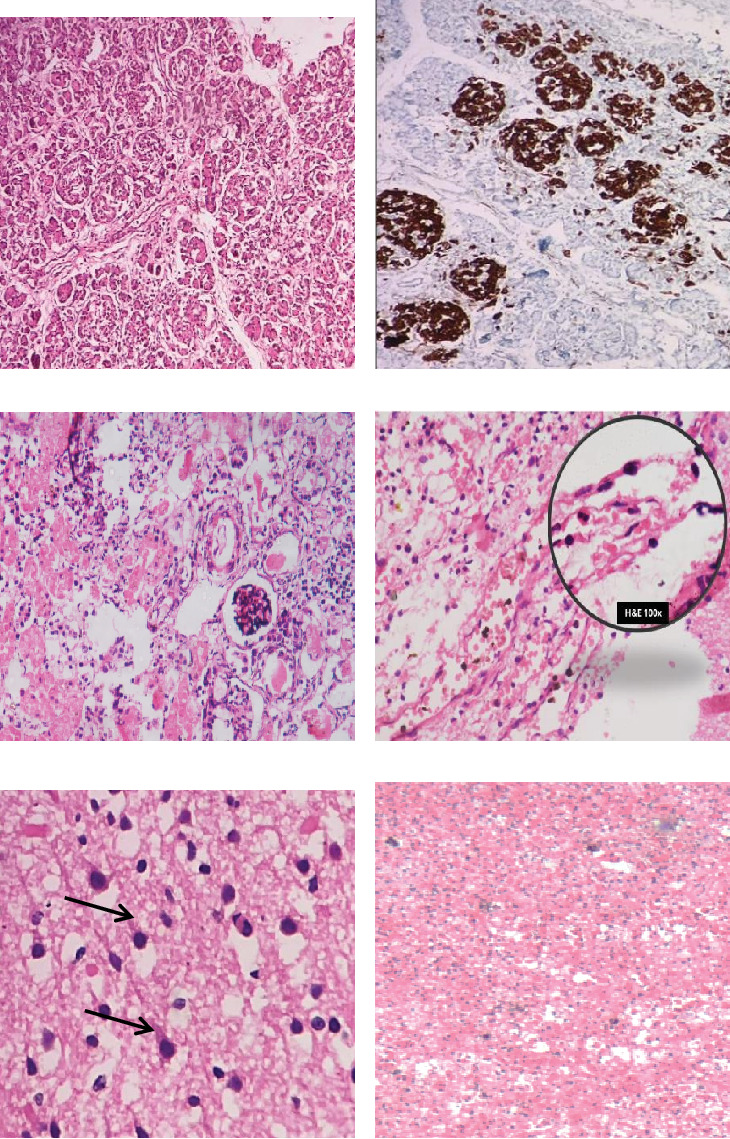
(a) Nodular hyperplasia of isle cell clusters, including ductuloinsular complexes and the same is highlighted on IHC with (b) synaptophysin. (c) Features of acute tubular necrosis were seen in the renal parenchyma in the form of simplification of tubular epithelium with blebbing of apical cytoplasm with increased eosinophilic staining. Tubular lumens filled with sloughed off necrotic tubular epithelial cells are also seen. The glomeruli are immature with a crown of podocytes. (d) Meninges show acute inflammatory infiltrate indicating meningeal inflammation, and the inset shows neutrophils at 100x. (e) The brain parenchyma reveals “red neurons” recognized by their shrunken cell body and intense cytoplasmic eosinophilia with complete loss of Nissl basophilia (black arrows). (f) Completely effaced out adrenal architecture with diffuse hemorrhage.

Following parental consent, a detailed autopsy examination was undertaken (Figures 1, 2, and 3).

## 3. Autopsy Findings (Rephrased)

External examination revealed well-formed male genitalia, with rigor mortis and postmortem lividity present. Intravenous cannulation marks were observed on both hands. There was no evidence of meconium staining over the skin or natural orifices. Anthropometric parameters were within the expected range for gestational age, and no gross congenital anomalies were detected.

A standard Y-shaped thoracoabdominal incision was performed, and internal organs were removed en masse. The heart weighed 22.2 g, exceeding the reference range for 38 weeks' gestation (15.1 ± 6.9 g). Grossly, there was cardiomegaly with a straightened left cardiac border, rounding of the right heart silhouette, and thickening of the interventricular septum, right ventricular wall, and left ventricular free wall. The cardiac chamber appeared narrowed, giving a banana-shaped configuration. Histopathology revealed myocardial fiber disarray, Y-shaped branching of myocytes, increased side-to-side junctions, and nuclear enlargement. Electron microscopy was performed to rule out mitochondrial myopathy; no abnormal subsarcolemmal mitochondrial aggregates were noted.

The right and left lungs weighed 20.6 and 22.2 g, respectively, both below the expected combined lung weight of 39.8 ± 11.1 g for this gestational age. On gross inspection, the lungs were solid and lacked crepitation. Microscopic evaluation demonstrated lung immaturity, absence of mature alveolar structures, prominent interstitial connective tissue, and medial thickening of pulmonary arteries. Numerous hemosiderin-laden macrophages (heart failure cells) were present, suggesting chronic pulmonary congestion. Pulmonary vascular changes, including medial hypertrophy and early plexiform lesions, were also noted.

The pancreas, situated within the C-loop of the duodenum, exhibited prominent nodular hyperplasia of islet cell clusters with accompanying ductuloinsular complexes.

The right and left kidneys weighed 26 and 23 g, respectively (reference: 19.4 ± 9.7 g combined for this gestational age) and appeared grossly unremarkable. Microscopy showed immature glomerular development with peripheral podocyte prominence and evidence of acute tubular injury, characterized by brush border loss and cytoplasmic eosinophilia.

The adrenal glands weighed 1.5 g (right) and 2.5 g (left), both below the expected combined adrenal weight of 5.8 ± 6.2 g. Microscopic sections showed near-total architectural effacement with extensive hemorrhagic areas.

The brain weighed 255, reduced compared to the average for 38 weeks' gestation (297 ± 69 g). Gross examination revealed a semisolid consistency with thickened, opaque meninges. Microscopic examination highlighted hypoxic–ischemic neuronal injury, evidenced by shrunken, eosinophilic neurons with pyknotic nuclei and central chromatolysis.

The liver and gallbladder together weighed 150 g, exceeding the expected average liver weight (91 ± 57 g). The cut surface of the liver appeared enlarged and congested. Microscopy revealed foci of extramedullary hematopoiesis.

The spleen weighed 3.5 g, which is slightly reduced compared to the reference range (7.2 ± 6.3 g). The external surface was normal, with congestion evident on the cut section.

Based on the autopsy findings, the likely cause of death was concluded to be hypoxic–ischemic brain injury with multiorgan dysfunction, likely precipitated by underlying cardiac hypertrophy, pancreatic islet cell hyperplasia, and lung immaturity.

## 4. Discussion

Pregnancies complicated by DM carry a significantly increased risk of adverse fetal, neonatal, and long-term outcomes. The extent and nature of these complications depend on several factors, including whether the diabetes is pregestational or gestational, the degree of maternal glycemic control, and whether the mother requires insulin therapy [5].

The Pedersen hypothesis remains central to explaining diabetic fetopathy. According to this concept, maternal hyperglycemia leads to fetal hyperglycemia due to unrestricted glucose transfer across the placenta. In the early stages of gestation, specifically before 20 weeks, the fetal pancreas is functionally immature, rendering the fetus incapable of mounting an insulin response. Consequently, the embryo is exposed to a hyperglycemic environment, which adversely affects cellular development.

After 20 weeks' gestation, as the fetal pancreas matures, the fetus begins to regulate its glucose levels independently, since maternal insulin does not significantly cross the placental barrier. Fetal hyperglycemia stimulates islet cell hyperplasia and hyperinsulinemia, setting the stage for metabolic disturbances and structural anomalies.

Proposed mechanisms suggest that excess fetal glucose disrupts arachidonic acid and myoinositol metabolism, promoting oxidative stress and mitochondrial damage. This cascade interferes with organogenesis by altering gene expression patterns, dysregulating cellular mitosis, and enhancing apoptosis—factors contributing to congenital malformations [6–8].

The risk of major congenital anomalies among IDMs is estimated at 5%–6%, with this figure rising to 10%–12% in insulin-dependent diabetic pregnancies. Frequently affected systems include the central nervous system, cardiovascular system, respiratory tract, musculoskeletal structures, and genitourinary tract.

Maternal diabetes also elevates the risk of preterm labor, operative deliveries, neonatal hypoglycemia, hyperbilirubinemia, and increased admissions to NICUs. Furthermore, complications such as pregnancy-induced hypertension, congenital anomalies, and metabolic disturbances have been documented extensively in large cohort studies.

Cardiovascular malformations are among the most critical structural defects, reported in 3%–9% of IDM cases. These include atrial and ventricular septal defects, transposition of the great arteries, coarctation of the aorta, double outlet right ventricle, and hypertrophic cardiomyopathy, as was seen in our case.

Intrauterine hypoxia, macrosomia, dysfunctional labor, and shoulder dystocia further increase the risk of perinatal asphyxia in IDM. Large-scale studies, including one with over 55,000 IDM deliveries, report a perinatal asphyxia incidence of approximately 1.1% [9].

Emerging diagnostic tools such as speckle-tracking echocardiography provide objective assessment of myocardial deformation, offering insights into systolic and diastolic function. Studies indicate that IDM, particularly those born to mothers with obesity and poor glycemic control, exhibit persistent myocardial dysfunction detectable for up to 40 days postpartum [10].

Maternal obesity (BMI ≥ 30 kg/m^2^) and elevated HbA1c levels (≥ 38 mmol/mol) correlate with subclinical myocardial dysfunction in IDM, emphasizing the importance of optimal maternal health prior to and during pregnancy.

In the largest autopsy series of IDM by Driscoll et al., involving 95 infants, hyperplasia of pancreatic islets was noted in 81%, cardiovascular defects in 16%, and gross congenital malformations in over 45% of cases. Interestingly, generalized organomegaly and gigantism were less frequently observed [11]. Hyaline membrane disease emerged as the predominant cause of death, accounting for 52% of fatalities.

A meta-analysis by Shu et al. highlighted the association between maternal diabetes and persistent pulmonary hypertension of the newborn (PPHN), estimating that 2.2%–20% of PPHN cases may be attributable to maternal hyperglycemia, consistent with pulmonary findings in our case [12].

The detrimental impact of maternal diabetes on fetal lung maturation is well established, attributed to impaired surfactant protein synthesis, abnormal tubular myelin development, and hyperglycemia-induced enzymatic suppression, leading to reduced phosphatidylglycerol levels [13].

IDMs face increased susceptibility to complications spanning the periconceptional, fetal, neonatal, and long-term periods. While multiple factors contribute to these adverse outcomes, maternal glucose control remains pivotal in minimizing risk.

In our case, significant cardiopulmonary hypertrophy, pancreatic islet cell hyperplasia, lung immaturity, and hypoxic brain injury were identified at autopsy, suggesting maternal–fetal glucose imbalance as the underlying pathophysiological mechanism.

Though postmortem diagnosis of neonatal hypoglycemia is challenging, characteristic gross and histological findings combined with maternal diabetic history should prompt consideration of glucose dysregulation as a contributory factor [14–16].

Despite advancements in molecular diagnostics, autopsy remains invaluable in determining causes of neonatal death. As highlighted in this case, autopsy findings can provide essential insights, guiding clinical practice and facilitating informed genetic counseling for affected families.

## Data Availability

The data that support the findings of this study are available on request from the corresponding author. The data are not publicly available due to privacy or ethical restrictions.
