# Supplementary material for: An Autopsy Case Report of an Infant Born to a Diabetic Mother, With Review of Literature—A Pandora's Box of Pathologies
Source: Case Rep Pathol. 2025 Oct 26;2025:6662921. doi: 10.1155/crip/6662921 (PMC12580034; doi:10.1155/crip/6662921)
Supplement: Supporting Information — Additional supporting information can be found online in the Supporting Information section. [file 6662921.f1.docx]

**CARE checklist**

| **Topic** | **Item** | **Checklist item description** | **Line** |
| --- | --- | --- | --- |
| Title | 1 | The diagnosis or intervention of primary focus followed by the words 'case report' | 1 & 4 |
| Key Words | 2 | 2 to 5 key words that identify diagnoses or interventions in this case report, including 'case report' | 68 |
| Abstract (no references) | 3a | Introduction: What is unique about this case and what does it add to the scientific literature? | 59 |
|  | 3b | Main symptoms and/or important clinical findings | 60, 61 |
|  | 3c | The main diagnoses, therapeutic interventions, and outcomes | 62, 63 |
|  | 3d | Conclusion What is the main 'take-away' lesson(s) from this case? | 66, 67 |
| Introduction | 4 | One or two paragraphs summarizing why this case is unique (may include references) | 62-67 |
| Patient Information | 5a | De-identified patient specific information | 87 |
|  | 5b | Primary concerns and symptoms of the patient | 88-89 |
|  | 5c | Medical, family history | 89 |
|  | 5d | Relevant past interventions with outcomes | - |
| Clinical Findings | 6 | Describe significant physical examination (PE) and important clinical findings | 87-99 |
| Timeline | 7 | Historical and current information from this episode of care organized as a timeline | - |
| Diagnostic Assessment | 8a | Diagnostic testing (such as PE, laboratory testing, imaging, surveys) | 92 |
|  | 8b | Diagnostic challenges (such as access to testing, financial, or cultural) | - |
|  | 8c | Diagnosis (including other diagnoses considered) | 139-141 |
|  | 8d | Prognosis (such as staging in oncology) where applicable | - |
| Therapeutic Intervention | 9a | Types of therapeutic intervention (such as pharmacologic, surgical, preventive, self-care) | 96-100 |
|  | 9b | Administration of therapeutic intervention (such as dosage, strength, duration) | 96-100 |
|  | 9c | Changes in therapeutic intervention (with rationale) | - |
| Follow-up and Outcomes | 10a | Clinician and patient-assessed outcomes (if available) |  |
|  | 10b | Important follow-up diagnostic and other test results | 139 |
|  | 10c | Intervention adherence and tolerability (How was this assessed?) | 92-100 |
|  | 10d | Adverse and unanticipated events | 97-100 |
| Discussion | 11a | A scientific discussion of the strengths AND limitations associated with this case report | 197-202 |
|  | 11b | Discussion of the relevant medical literature with references | 168-187 |
|  | 11c | The scientific rationale for any conclusions (including assessment of possible causes) | 139-141 |
|  | 11d | The primary 'take-away' lessons of this case report (without references) in a one paragraph conclusion | 200-202 |
| Patient Perspective | 12 | The patient should share their perspective in one to two paragraphs on the treatment(s) they received | This is an autopsy case report. Intervention received is mentioned in lines 92 – 100. |
| Informed Consent | 13 | Did the patient give informed consent? Please provide if requested | Yes |
